# Can economic evaluation in telemedicine be trusted? A systematic review of the literature

**DOI:** 10.1186/1478-7547-7-18

**Published:** 2009-10-24

**Authors:** Trine S Bergmo

**Affiliations:** 1 Norwegian Centre for Integrated Care and Telemedicine, University Hospital of North Norway, N-9038 Tromsø, Norway

## Abstract

**Background:**

Telemedicine has been advocated as an effective means to provide health care services over a distance. Systematic information on costs and consequences has been called for to support decision-making in this field. This paper provides a review of the quality, validity and generalisability of economic evaluations in telemedicine.

**Methods:**

A systematic literature search in all relevant databases was conducted and forms the basis for addressing these issues. Only articles published in peer-reviewed journals and written in English in the period from 1990 to 2007 were analysed. The literature search identified 33 economic evaluations where both costs (resource use) and outcomes (non-resource consequences) were measured.

**Results:**

This review shows that economic evaluations in telemedicine are highly diverse in terms of both the study context and the methods applied. The articles covered several medical specialities ranging from cardiology and dermatology to psychiatry. The studies analysed telemedicine in home care, and in primary and secondary care settings using a variety of different technologies including videoconferencing, still-images and monitoring (store-and-forward telemedicine). Most studies used multiple outcome measures and analysed the effects using disaggregated cost-consequence frameworks. Objectives, study design, and choice of comparators were mostly well reported. The majority of the studies lacked information on perspective and costing method, few used general statistics and sensitivity analysis to assess validity, and even fewer used marginal analysis.

**Conclusion:**

As this paper demonstrates, the majority of the economic evaluations reviewed were not in accordance with standard evaluation techniques. Further research is needed to explore the reasons for this and to address how economic evaluation in telemedicine best can take advantage of local constraints and at the same time produce valid and generalisable results.

## Background

There is a growing interest in shifting health care delivery from costly secondary care hospitals to community settings bringing care 'closer to home' for patients [1]. Telemedicine has been advocated as an effective means to deliver health services to remote communities. Telemedicine can be defined as 'distance medicine' using information and communication technologies (ICT) to examine, monitor, treat and care for patients over a distance. Different applications can be used both within and between all kind of health care institutions as well as to monitor and provide support to patients living at home. Telemedicine can also be used to improve the chain of care and may involve complex delivery systems that employ a mix of technologies in addition to innovative clinical processes [2]. Recent telemedicine applications encompass activities such as remote consultations in a wide range of specialities ranging from dermatology and cardiology to psychiatry. Other examples are transmission of echocardiograms, blood glucose levels, and x-rays; provision of accident and emergency expertise to remote locations; remote support and monitoring of patients undergoing dialysis; remote fetal monitoring; support and care to elderly people or to patients with chronic conditions living at home. In this review, telemedicine refers to technologies used in direct patient care such as real-time videoconferencing and store-and-forward applications. The former involves live sound and picture. The latter involves asynchronous transmission of medical data such as video films (ultrasound), still- images (x-rays, photographs of skin lesions and other static images) and sound files (heart murmurs).

As with any other form of health care technology there is a need to assess effectiveness, efficiency and safety before it is brought into wider use [3]. Telemedicine evaluation should first ensure that the technology is safe and generates as much benefit as conventional care. If using the technology produces equal or better health or quality of life, the next step is to analyse differences in costs (one should note, however, that services could generate less benefit at lower cost and still be considered cost-effective). Cost savings and other benefits of telemedicine are often suggested by the logic of its impact on health care delivery and by the optimistic promise of ICT in general [2]. Systematic information on costs and consequences has been called for to support decision-making both in order to control health care spending; and to document value for money to facilitate reimbursement of telemedicine activities.

Economic evaluation is a set of formal analytical techniques that provide systematic information about costs and benefits of alternative options, and can thereby assist in priority-setting [4-7]. If economic evaluation is advocated as an important support tool for decision-making, it seems appropriate to monitor the quality of these evaluations. This will also provide a better understanding of economic evaluation applied to telemedicine. Several systematic reviews have found little evidence that telemedicine is cost saving [8-10]. Reviewers noted few rigorous evaluations and even useful studies had some questionable use of standard techniques for economic evaluations [2,11-13]. High-quality evidence of the technologies' impact on patients' health and well-being is also lacking [3,9,14].

Other systematic reviews investigating the cost effectiveness of telemedicine found that most studies evaluated benefits in terms of cost savings with no assessment of changes in the benefits for patients [8,13]. This review explores whether more recent evaluations have included patient outcomes and it analyses only full economic evaluations, i.e. evaluations where both costs (resource use) and outcomes (non-resource consequences) have been measured and valued.

The purpose of this paper is to provide a detailed review of full economic evaluations of telemedicine use. This qualitative assessment has three objectives: to provide detailed information on evaluation approaches and applied methods; to assess the quality of the evaluations and their potential contribution to priority setting; and to discuss the applicability of best-practice methods to telemedicine settings. The focus is on exploring the following aspects: clarity of study objective; adequacy of comparison; choice of study perspective and design; measuring and valuation of costs and outcomes; reporting of data sources; handling of uncertainty; presentation of results.

## Methods

### Selection criteria

A systematic literature review was conducted to identify all published articles describing economic evaluation of telemedicine use. It is clear from the literature that telemedicine encompasses a wide range of different technologies and services used in a variety of medical settings. In this review telemedicine refers to the use of information and communication technologies in direct patient care, i.e. it covers only aspects in which the patient is directly involved and the patient and provider are separated by distance. Services that did not require encounters between patients and providers, such as radiology and pathology were therefore excluded. Technologies of interest were audio and visual (i.e. videoconferencing) or just visual (still-image telemedicine) or audio and data transfer (monitoring) excluding telephone calls without any transfer of medical data, traditional e-mails, and information and support sites on the Internet. This restriction has been imposed to make the review process more manageable and corresponds to a previous review [3].

Only articles written in English from 1990 to 2007 were included. These must have been published in peer-reviewed journals. This excluded books, HTA reports and unpublished materials. The articles included were full economic evaluations, i.e. they undertook a comparative analysis of both costs as resource use and outcomes in form of non-resource consequences of at least two alternatives, (or one if it was a cost-benefit study). The non-resource consequences typically refer to the effect that alternative interventions have on peoples' health status. The articles included described either applied studies, i.e. they generated primary data, or studies in which secondary data was modelled. Cost analyses where the consequences of two interventions were assumed to be identical across the alternatives were excluded. In this review, evaluations using outcome measures such as successful telemedicine consultations, travels avoided, hospitalisations avoided and other avoided costs have been categorised as cost analyses and excluded.

### Search strategy

A literature search was undertaken using the British National Health Service Economic Evaluation Database (NHS EED), PubMed, PsychInfo, Embase, CHINAL, Ovid Nursing, British Nursing Index, ISI-Web of Knowledge, EconLit and Telemedicine Information Exchange. The two main journals in the telemedicine field, 'Journal of Telemedicine and Telecare' and 'Telemedicine Journal and E-health', in addition to the reference lists in the retrieved articles and existing reviews, have been manually searched.

The search strategy included the Thesaurus terms "telemedicine" (including all subheadings) and "videoconferencing" (as a single term) and various other relevant text words. This search strategy was combined with Thesaurus "Costs and Cost-analyses" (including all subheadings). The latter is one hierarchical level above "Cost-benefit analysis" in the sub-tree, has a high sensitivity and has been recommended as a search strategy to identify economic evaluations [15,16]. When medical subheading terms were not available, the term was searched as a keyword. The search strategy in the non-medical database included text searches using "telemedicine", "telehealth", "remote consultations", "videoconferencing", "teleconsultations" and "telecare", while "cost", "cost analysis", "cost-effectiveness" and "economics" were included in the Telemedicine Information Exchange search. As a supplement, free-text searches in the medical databases with the search terms mentioned above were also conducted to include the more recent economic evaluations.

### Review process

Selection of relevant publications was based on information found in the abstracts. Full-text articles were retrieved when the abstract indicated analyses of both costs and non-resource consequences. Full-text was also retrieved for closer inspection if the abstract did not provide clear indication of the content. All abstracts and full-text articles were read by the author.

Data were extracted from the full-text articles based on international benchmarks on conducting and reporting of economic evaluations [7,17]. Such recommendations often have forms as checklists and these identify key parameters that one may expect to find in well-executed studies. The main parameters used to assess the articles reviewed in this paper were clarity of study objective, adequacy of comparison, choice of study perspective and design, transparency in the measurements and valuation of costs and outcomes, reporting of data sources, handling of uncertainty, and presentation of results. These evaluation criteria have been chosen because they reflect the main challenges in telemedicine evaluations reported in the literature [2,18]. In addition, details on the intervention type, medical field, type of analysis, and key findings have been collected (see Additional file 1). The complete data table is obtainable from the author on request.

## Results

### Summary of economic studies in telemedicine

The literature search identified 779 abstracts which were reviewed for relevance. For 89 of these, full-text articles were retrieved. After review, 33 articles were found to meet the inclusion criteria, i.e. they measured both costs and consequences of telemedicine.

Additional file 1 provides an overview of the studies included in this review. The articles covered several different medical specialities ranging from dermatology and cardiology to psychiatry. The use of telemedicine was most common in diabetes care (n = 6) and cardiology (n = 6). Almost half of the studies evaluated telemedicine in home care (n = 15), the rest evaluated services provided either in secondary care or between primary and secondary care institutions. The services provided used a variety of different technologies ranging from store-and-forward telemedicine (n = 16) to real-time videoconferencing (n = 7) and a combination of the two (n = 10) (see Additional file 1 for details). One third of the studies (n = 11) were published in the two main telemedicine journals: 'Journal of Telemedicine and Telecare' and 'Telemedicine and e-Health', while the others were published in a variety of medical and health research journals.

### Study objective and comparators

A clear description of the study objective together with information on the comparators is vital for assessing the quality of an economic evaluation. This will provide the reader with information both on the aim of the evaluation and on the decision-making context. In the majority of the studies (n = 28) the objectives were stated in a clear and unambiguous manner and mainly addressed choice of techniques as well as cost-effective resource allocations. One study stated clearly that the main objective was to test the feasibility of the technology [19]. All but six studies [20-25] provided a clear description of the comparators and sufficient information on the study groups (who received what). Almost all studies (n = 32) compared telemedicine to at least one alternative without telemedicine. This was referred to as a "usual care or current alternative" in some kind of on-site encounter. This is a relevant and appropriate comparator as it reflects the conventional approach to patient management. One study compared two different telemedicine techniques which appeared appropriate in their context [26]. In six studies telemedicine was analysed as a complement to traditional home care [21,27-31], while in another three, it was unclear if telemedicine was provided in addition to or instead of usual care [23-25].

### Study design and perspective

The majority of the studies reviewed were trial-based or observational and included randomised controlled trials (n = 13), non-randomised trials (n = 2), case control studies with the controls receiving standard care (n = 3), before and after studies (n = 3), case series (n = 1) and crossover trials with one group (n = 2) (see Additional file 1 for details). One study used two groups, but no information on study design was provided [25], one study used matched controls [32] and one study compared before and after results from different patients, which the authors acknowledged as a limitation [33].

Six of the evaluations were decision-modelling studies using secondary data to analyse costs and consequences (see Additional file 1). Five of the studies involved a literature search to obtain the primary studies [34-38], while one study used results from one single study to derive the effectiveness evidence [39]. None of the studies provided information on the search strategy, the inclusion criteria or the method used to extract relevant data (one study reported a MedLine search [37]). The number of primary studies ranged from four to 14. Three studies did not provide information about the model used [35,37,39], two used decision-tree modelling [36,38] and one used a Markov model [34]. The modelling studies analysed telemedicine as a means to support diabetes care [34,38], pre-hospital care [35,37], dermatology [39] and paediatric ophthalmology [36].

Two studies reported use of a societal perspective [35,40], eight reported a health provider perspective [27,33,34,36-39,41] and two reported a combination of health provider and patient perspectives [42,43]. One of the studies which reported a societal perspective only included direct health care costs [40] while the other also included costs borne outside the health care system (police, lawsuits and insurance) [35]. Two thirds of the studies (n = 21) did not explicitly report the study perspective.

### Costing

The costs of telemedicine can be divided into two broad categories: health care costs and non-health care costs. Direct health care costs refer to the physical health resources required to produce a specific health service or programme. Non-health care costs are those outside the health care sector, for example time costs such as production loss or lost leisure time, travel costs and costs associated with childcare. All studies in this review measured direct health care costs. These included costs related to investment, installation, call costs, personnel costs, and other health care costs. In addition, travel costs for health care personnel were included if a visiting service or home care was the alternative to telemedicine (n = 8) [27-29,31,32,34,39,44]. Fewer than half of the studies (n = 11) estimated private travel costs and even fewer estimated time costs as production losses (n = 8) (see Additional file 1 for details). For the majority of the studies (n = 22) the patient did not travel or the health provider (for example federal agencies in the US) paid for travel (n = 3) [29,34,39]. Five studies provided no [45] or very limited information [24,30,32,37] on the costs included, and in almost half of the evaluations (n = 14) the costing method was unclear. Fifteen studies provided details on resources consumed in physical units and reported prices or unit costs separately from quantities. Five of these reported only certain costs and quantities separately.

The studies reviewed appeared to have used some combination of an 'ingredients' approach and an 'activity' based approach to costing. The former is a costing method where every cost item is broken down into its underlying components, while the latter does not and refers to, for example, the costs of a hospital bed-day without any further information. An 'ingredients' approach was used to calculate the cost of providing the telemedicine service or the intervention, while 'activity' based costing was mainly used to calculate hospital costs and the costs of home care visits. This combination of 'ingredients' and 'activity' approaches is common in economic analyses in health care in general [46]. Three studies used an 'activity' approach only [24,27,37].

In one third of the studies (n = 10) some of the cost items were calculated using charges or reimbursement fees [19,22,24,25,27,33,34,41,47,48]. These were mainly hospital in-patient costs, costs of outpatient visits and costs of home care visits. One study estimated the hospital costs as a cost-to-charge ratio [33], which is a method that calculates costs using prices adjusted for excess profit and is mainly used in the US [6]. For the majority of the studies (n = 18) it was not possible to determine how hospital costs had been valued. One study reported the use of an anecdotal costing method and estimated hypothetical hospital savings [49]. The costs of providing the telemedicine service were calculated using market prices and average staff wages. Time costs for patients and relatives were valued using different shadow prices ranging from actual loss of earnings [50], to estimates of production loss using the friction method [51]. One study estimated lost earnings, lost overtime and lost unpaid work [43].

Tariffs or user fees do not necessarily reflect the true opportunity costs of resource use. Only two studies addressed the potential limitation of using charges to estimate the costs of an intervention [27,33]. None of the studies assessed telemedicine costing in relation to production capacity or cost sharing. Only three studies reported calculation of marginal costs [21,41,50].

### Measuring the consequences

Additional file 1 shows that there is considerable variation in the measures used to assess the non-resource consequences in economic evaluations of telemedicine. The effectiveness measures presented in the studies ranged from impacts on process to final outcomes. The measures varied from diagnostic accuracy, blood glucose levels, anxiety and depression levels, physical capacity and health-related quality of life (HRQL) to life-years gained (LYG) and quality-adjusted life-years (QALYs).

Examples of process measures presented in the studies include diagnostic accuracy and agreement in reading of transmitted data (n = 5), medication compliance (n = 2), time spent on diabetes care for both patient and professionals (n = 1), number of days to independent pouch change after abdominal surgery and colostomy (n = 1) and length of stay (n = 3) (see Additional file 1 for details). Examples of disease-specific surrogate measures used were blood glucose levels, hypoglycaemic events, percentage reduction in wound size and body mass index. These are all measures that can be related to future health status. Seven studies used generic health-related quality of life measures (SF-36 and EQ-5D), of which two studies also used disease- or group-specific QoL-instruments [42,52]. Other generic outcome measures employed in the reviewed studies were return to activities of daily living (n = 3), mortality in the form of death avoided or life-years gained (n = 5) and QALYs gained (n = 4). One study measured disability-adjusted life-years gained [22]. The majority of the studies (n = 24) used multiple outcome measures to evaluate effectiveness. Of the trial-based studies, three used secondary data to measure effectiveness, i.e. the effectiveness data was reported in different articles [23,43,50].

### Other technical issues

The studies reviewed varied with regard to whether they reported their data sources or not. Four of the studies did not report data sources for either costs or consequences [19,25,32,49]. All the remaining studies reported the sources for the effectiveness data (n = 29). These were, for example, questionnaires, existing literature, patient chart records, case notes, and other clinical databases. The sources for the cost estimates were explicitly reported in most of the studies (n = 25). These were hospital accounting systems, project diaries, patient charts, case notes and official databases. Four of the studies reporting data sources for the effectiveness data did not report sources for the cost data [30,39,45,47]. The modelling studies used existing literature, official statistics, and databases; and some included expert and author opinions (see Additional file 1 for details).

Fewer than half of the studies (n = 13) [22,23,31,34-40,42,51,53] included sensitivity analysis to assess the robustness of the findings to variability in parameters or model inputs. One-way analyses were performed in nine studies [22,23,36-39,42,51,53], multi-way sensitivity analysis was performed in one study [35] and both types of analyses were performed in three studies [34,40,43]. The majority of the studies (n = 23) used some kind of statistical analyses to calculate confidence intervals around point estimates for the outcome measures. Three employed non-parametric bootstrapping to estimate confidence intervals around mean costs [42,43,51]. In four studies costs were treated deterministically with no need to account for the variability in the cost estimates [19,21,32,39]. Of the total studies, less than one third (n = 10) applied sensitivity analysis, together with appropriate statistics to address the variability in both the effectiveness measure and the costs (where these were not treated deterministically).

Costs and consequences were summarised in a cost per effect measure in less than one third of the studies (n = 9) [22,34,35,37-40,45,51]. Incremental cost-effectiveness ratios were calculated in five studies [34,38,39,45,51]. Five studies were CUAs while the rest were CEAs. Of the latter, four reported use of cost-minimisation techniques (CMA), and 19 took disaggregated forms and employed a cost-consequence framework (CCA) (see Additional file 1 for details). The latter lists all the different outcomes together with the costs instead of using a one-dimensional outcome measure. The costs in these studies were calculated as total costs, annual costs or unit costs. The latter were most frequently presented as a cost per patient or a cost per visit/session (n = 15).

It is important to consider whether the costs and consequences of interventions and their alternatives can be adapted from one context to another. Only six studies explicitly addressed the issue of generalisability to other settings [21,34,35,38-40]. Another two studies included extensive sensitivity analyses [43,51], which improved the external validity. Limitations are however acknowledged in the majority of the studies (n = 18) with statements that aspects such as a retrospective design, too small sample size, the use of charges and fees, a high drop-out rate and wide confidence interval might limit the validity of the results.

## Discussion

This review shows that economic evaluations in telemedicine are highly diverse in terms of both the study context and methods applied. The articles covered several medical specialities and analysed telemedicine in home care as well as in primary and secondary care settings using a variety of different technologies. Most studies used multiple outcome measures and analysed the effects using disaggregated cost-consequence frameworks.

One of the main arguments for using telemedicine is that these technologies have the potential to reduce health care costs and enable cost-effective resource allocation. This review identified 33 full economic studies measuring both costs and consequences. The fact that only articles in English and published in peer-reviewed journals (to provide some basic quality control) were included is recognised as a limitation.

Even if the evidence base for telemedicine decisions is alarmingly scarce, to simply increase the number of economic studies in telemedicine must be considered in relation to a number of concerns highlighted by findings in both this and other reviews [2,11]. Decision-makers need to be confident that the economic studies in telemedicine are consistent and reliable. Only eight studies [34,36,38-40,42,50,51] had addressed all the key issues described in the Method section (a clear study objective, adequate comparison(s), reporting of study perspective and design, transparent measurements and valuation of costs and outcomes, reporting of data sources, addressing of uncertainty and clear presentation of the results). The remaining studies had several shortcomings primarily concerning technical aspects and reporting of results. Objectives, study design and choice of comparators were mostly well reported. The majority of the studies lacked information on the perspective, few used general statistics and sensitivity analysis to assess validity, and even fewer used marginal analysis. The effectiveness measures appeared more consistent and well reported than the costing, which was often unclear. Some of these methodological issues will be addressed in more detail below, along with implications and recommendations.

### Measuring effectiveness and patient safety

In some settings, telemedicine services are replacing traditional in-person encounters between patients and health care personnel. In these situations disease- or case-specific measures are sufficient to estimate the relative effectiveness of telemedicine versus conventional approaches to patient management. These measurements can even be at an ordinal level. If specific outcome measures show equal or better patient outcomes than usual care, then the next step is to assess the differences in costs using standard costing techniques. This approach to telemedicine evaluation will however limit generalisability and make it impossible to compare or synthesise results from evaluations with different disease-specific outcome measures.

Telemedicine can also be used to provide a completely new service alongside traditional care such as monitoring of chronic conditions for patients living at home. In these situations telemedicine is provided in addition to traditional home care and could potentially improve patients' health. For example, if investing in telemedicine costs more and is more effective, the decision-maker would need information on how much more beneficial it is for the costs involved. To be able to compare this with other services and programmes generic health status measurements such as QALYs or LYG are required.

Consistency in effectiveness measures has important implications for the usefulness of cost effectiveness results to decision-making [5]. This review found outcome measures ranging from diagnostic accuracy, blood glucose levels, and quality of life measures to QALYs. Diagnostic accuracy was frequently used together with surrogate measures. The former will ensure quality of the transmitted information. The latter is related to future health and can be viewed as inputs into a health production function [54]. If the objective of using telemedicine technologies in diabetes care is to reduce and stabilise blood glucose levels, it seems appropriate for the endpoint to measure blood glucose levels. On the other hand, it can be difficult to interpret cost-effectiveness in terms of a specific cost per reduction in blood glucose level. Another example is if the decision is whether to invest in telemedicine in dermatology or not, the consequence measure employed can be intermediate and case-specific, i.e. related to skin problems. While these examples are acceptable for assessing technical efficiency, (i.e. how to produce a given level of health outcome at least cost) this will not help in deciding how to allocate resources across programmes. In these situations, generic health measures are required to allow for comparison between the two such as LYG or QALYs. Few studies used such generic health measures. One reason for this may be that these are not sensitive enough to detect small changes in health outcomes which telemedicine services most likely produce. Another reason may be that most economic evaluations have been undertaken to justify decisions within clinical areas and to support reimbursement and payment systems, and not as a basis for broader decision-making.

### Inclusion of non-health care costs

In the economic evaluation of telemedicine, the distinction between costs incurred within or outside the health care system seems particularly relevant. One argument for promoting telemedicine is that the technology has potential to generate cost savings that are mainly outside the health care system. In many health care systems, it is the patients who pay the travel costs that telemedicine eliminates, while the time savings are often valued as production changes. The stakeholders bearing the costs may differ from those experiencing the benefits, which in most cases are patients and employers. To include non-health care costs however requires the measurement of opportunity costs to society and not just the opportunity costs as health benefits forgone. These savings are excluded if one adopts a health provider perspective. It is therefore important to be clear about the viewpoint chosen for the analysis and how this affects the results. This will also help the reader judge whether all the relevant costs are included. Few of the reviewed studies did explicitly report their study perspective. It was however possible to infer the viewpoint in most studies. Not reporting the perspective explicitly is a common failing in economic evaluations in general, even if the costs included are clearly presented [5].

### Lack of transparency in costing methods

The costs included in the reviewed studies were mainly direct health care costs and direct non-health costs. The former referred to the costs associated with providing the alternatives while the latter were time costs and travel costs. In some studies however, it was impossible to tell the type and magnitude of the costs items included. More than half of the studies provided limited or unclear cost information and did not present unit cost data alongside resource data. This could make it difficult for the reader to assess whether all appropriate costs were included and to judge whether the cost results could be adapted to their own setting. It also appeared that in some studies the total cost per visit differed from the cost per patient. Another challenge is the inclusion and valuation of time costs. Very few studies provided any reasons for including time costs. For example, one study gave no rationale for including time costs for patients or their families and friends [21]. Another study included lost work and lost overtime in addition to lost unpaid time [43]. A third study included time costs for the patients in a sensitivity analysis [39]. Presenting non-health care costs separately from health care costs in a sensitivity analysis may seem more appropriate, implying that the former may have less direct impact in decisions. In practice, patients may already be off work because of their health condition, leaving the actual production loss unchanged. There is however, a low level of agreement in the literature about whether to include productivity changes or not [7,55,56].

A clear study objective and relevant comparators are important, since economic evaluation is concerned with measuring the marginal costs and benefits relative to their highest valued alternative use [55]. In some analyses it was unclear whether telemedicine was provided in addition to or as a substitute for traditional health care. In one of the studies, it appeared that telemedicine visits were provided in addition to regular home visits without the costs of the latter being accounted for in the telemedicine alternative [31]. To compare the cost per visit for telemedicine and home visits will be misleading if these costs differ from the total cost per patient. On the other hand, in a real clinical situation it seems unlikely that telemedicine could be a complete substitute for in-person encounters; some combination of the two will probably be required. Similarly, analysing two different telemedicine alternatives without comparison to a current alternative will not produce relevant information if the objective is to decide whether to invest in or reimburse telemedicine services.

To calculate the cost of, for example, an outpatient consultation or a hospital bed-day is often time consuming and costly. Since most evaluations operate within budget constraints, it is easier to use readily available cost data such as charges or tariffs. Even if these are often used to calculate hospital costs [6] in most health care systems they are only financial parameters with no relation to actual resource consumption. In health systems with both tariffs and lump sum financing, tariffs may cover only part of the total cost of an activity. Hospitals are often multi-product organisations with a high degree of cross-subsidies and tariffs may therefore only be vehicles for funding activities. Only a few studies acknowledge that using charges could limit the validity of their findings.

### Generalisability

One of the main challenges in all economic evaluations is to balance the need for internal validity against the ability to generalise results to other settings. One third of the studies reviewed were evaluations alongside randomised controlled trials (RCT). RCT is a design with stringent criteria for selecting participants and a strict compliance to the study protocol, minimising potential bias. The RCT may however not be a suitable design for evaluating telemedicine, which is highly sensitive to local conditions. For instance randomisation will not ensure that the intervention is separated from the context [2], i.e. that the distance between the sites and other local costs are equally distributed in two groups. It is also common in telemedicine research to allow for self-selection. A naturalistic design or decision modelling might be more appropriate. The modelling studies in this review provided few details about both the model employed and the criteria used to ensure the validity of the primary studies, making it difficult to assess the robustness of the estimate measures. On the other hand, extensive sensitivity analyses improved the external validity of these studies.

Whether to use trial-based data or modelling studies should be seen in relation to the objective of the study and the viewpoint of those who are expected to use the results. Some researchers argue that trial-based studies are superior, while others view them as complementary rather than alternative approaches [4]. Decisions about whether to invest in telemedicine should ideally be based on a synthesis of all available data and not just on a single trial. In telemedicine research, however, there are few studies of good quality available in the different medical fields [2,14], which limits the use of decision modelling.

Researchers should comment directly on the transferability of their findings [57]. Some studies did acknowledge limitations, but these were mainly related to internal validity such as a low sample size and wide confidence intervals. It is also important to address situation-specific features that could influence the results, such as local treatment procedures or organisational structures. Costs and outcomes of interventions are always associated with some degree of uncertainty. Uncertainty may be due to sampling variation in estimates of both costs and outcomes and it may also be related to the economic model and the evaluation process [58]. In addition to highly different contexts and local settings, other parameters such as perspective, assumptions regarding cost and outcome identification, measurements and valuation may affect the results. Over half of the studies did not include sensitivity analysis. This limits the usefulness of the cost-effectiveness data as a basis for health system decision-making.

Presenting the cost and outcomes disaggregated in cost-consequence analyses (CCA) has been suggested as a useful systematic framework in evaluating telemedicine [59]. More than half of the reviewed studies presented their results using this framework. CCA lists all the different outcomes together with the costs instead of using a unidimensional outcome measure. This partly overcomes the challenge of deciding on a common outcome measure in different telemedicine settings, but severely limits generalisability. Multidimensional presentation of the consequences can also be difficult to interpret, especially if the different outcome dimensions move in different directions [54]. Another challenge is how to value, for example, a two-week reduction in waiting time if the consequences are presented in a descriptive manner. Other typical benefits claimed for telemedicine that are difficult to value would include improved quality of service, speed of service and transfer of skills. More research is needed to address these aspects. Hailey (2005) argues that the one of the immediate needs in economic evaluation of telemedicine is to improve clarity in the reporting of the economic findings [18].

## Summary and conclusion

For studies in telemedicine, as in economic evaluations of other medical technologies, it is important to be clear about the objective, comparators, perspective and study design. This will make the study transparent and help readers to decide whether results can apply to their own settings. Measuring and valuing the costs can be a major challenge, especially if the telemedicine services involve a mix of complex delivery systems and technologies. Aspects that need extra attention include how to handle shared resources, production capacity, marginal costs and the use of salaries and charges as proxies for opportunity costs. For example, one problem with using hospital accounting systems and charges is that some resources that are used but not billed may be overlooked. Different costing patterns may also make results from one study less adaptable to other settings. Measuring and valuing the consequences is another key challenge in the evaluation of telemedicine. Whether to use disease-specific or generic tools to measure the consequences should be seen in relation to the objectives and the type of services provided. If telemedicine technologies are employed to replace an in-person consultation, disease-specific instruments can be applied to ensure that the benefits for the patients are equivalent or better to that of conventional care. On the other hand, if telemedicine is provided in addition to existing traditional services for example in home care, other more generic health measures are more appropriate.

As this paper demonstrates, few economic evaluations of telemedicine can be trusted to provide reliable information for decision-making. The majority of the evaluations reviewed were not in accordance with standard evaluation techniques and still have a long way to go before decision-makers can rely on them to produce valid and reliable cost-effectiveness data. Such improvement refers primarily to technical aspects and reporting of results. Given the differences in decision problems, local settings and the range of analytical choices, it is not surprising that there is considerable variation in economic evaluations of telemedicine. Further research is needed to explore how much of this variation can be justified and accepted in telemedicine decision-making. It is also important to address how economic evaluations of telemedicine can best take advantage of local constraints and geographical heterogeneity and at the same time produce valid and reliable results.

## Competing interests

The author declares that they have no competing interests.

## Supplementary Material

Additional file 1 **Summary of the published economic evaluations in telemedicine from 1990-2007**. The file represents the table with details on author(s), year of publication, type of intervention, medical field, study design, type of analysis, effectiveness measure, costing, data sources and key findings for all included papers. Click here for file
