# Supplementary material for: Can economic evaluation in telemedicine be trusted? A systematic review of the literature
Source: Cost Eff Resour Alloc. 2009 Oct 24;7:18. doi: 10.1186/1478-7547-7-18 (PMC2770451; doi:10.1186/1478-7547-7-18)
Supplement: Additional file 1 — Summary of the published economic evaluations in telemedicine from 1990-2007. The file represents the table with details on author(s), year of publication, type of intervention, medical field, study design, type of analysis, effectiveness measure, costing, data sources and key findings for all included papers. [file 1478-7547-7-18-S1.DOC]

Additional file 1. Summary of the published economic evaluations in telemedicine from 1990-2007

| Author/Year | Intervention | Design | Measures of effectiveness | Costing | Data sources | Key findings | |  |
| --- | --- | --- | --- | --- | --- | --- | --- | --- |
| Aoki et al.  2004 [1] | Eye examination of prison inmates via TM | Decision-analytic modelling (CUA) | QALYs based on one study | Direct health care costs including publicly financed travel costs | Published studies, official reimbursement and transportation fees | TM dominates for the reference case subject and the simulation showed a cost-effective service |  | |
| Auerbach et al. 2006 [2] | TM device for pre-hospital traffic accident emergencies | Decision-analytic modelling (CEA) | LYG | Direct health care costs, non- health care costs and production loss | Published studies, accident databases authors’ opinions and administrative statistics and databases | TM may save lives, but at high costs. The cost-effectiveness ratio was €250 000 per LYG |  | |
| Barnett et al. 2007 [3] | Diabetes home care via TM | Before and after study (CUA) | QALYs based on SF36V | Direct health care costs, no details provided | Questionnaires, costs not reported | Mean ICER was $60 000 and within the range for cost-effectiveness |  | |
| Biermann et al. 2002 [4] | Diabetes home care via TM | RCT (CMA) | Blood glucose levels, time spent on diabetes care and patient satisfaction | Direct health care costs, travel and time costs for patients | Patient chart records and questionnaires (scenario cost comparison) | No significant differences in blood glucose level, reduced costs for TM |  | |
| Bohnenkamp et al 2004 [5] | Home support for ostomy care via VC | Non-randomised trial with two groups (CCA) | Average number of days to independent pouch change | Direct health care costs, no details provided | Questionnaires, costs not reported | No significant difference in effectiveness and equal costs, TM group had more total visits |  | |
| Breslow  et al. 2004 [6] | Intensive care staffing with TM | Before and after study of different patients (CCA) | Mortality and LOS | Direct health care costs | Observed in the study, hospital administration data | TM improved clinical outcome, reduced variable cost per case and increased marginal revenue |  | |
| Castillo-Riquelme  et al. 2004 [7] | Premature eye examinations via TM | Decision analytic modelling (CMA | QALYs based on survival and utility weights | Direct health care costs | Published studies and the authors’ opinions | Visiting nurse is a cost-effective alternative assuming equal accuracy |  | |
| Dawson et al. 1999 [8] | Home surveillance  of preterm labour | RCT (CCA) | Anxiety and depression, spontaneous labour and maternal satisfaction | Direct health care costs, travel and time costs for patients, their family and friends | Questionnaires and case notes, costs not reported | Equal clinical process in both groups and reduced cost for TM |  | |
| Dowie et al 2007 [9] | Paediatric cardiology  examinations via TM | Case control study (CCA) | Health related QoL (EQ-5D, QALIN and PedsQoL) | Direct health care costs including ambulance and travel costs for patients and their family | Questionnaires and case notes | Increased health related QoL for women, TM more costly for all patient, but less costly for babies due to ambulance transport |  | |
| Eron and Marineau 2007 [10] | Home treatment of serious infections via VC | Case control study (CCA) | Return to ADL | Direct health care costs | Not reported | TM improved return to ADL at reduced costs |  | |
| Ferris et al 2004 [11] | Gynaecologic examinations via two TM alternatives | Case series; one group underwent both alternatives (CMA) | Diagnostic accuracy | Direct health care costs | Observed in the study,  hospital administration data | Equal effectiveness of the two systems and computer based telecolposcopy costs less than network telecolposcopy |  | |
| Finkelstein  et al 2006 [12] | Home care via TM for chronic conditions | RCT with three groups (CCA) | Mortality, morbidity (measuring knowledge, behaviour and status) | Direct health care costs incl. travel costs for the home visits | Case notes, assessment tools and reimbursement fees | No difference in mortality, better ADL-levels for TM group and costs per visits cheaper for TM |  | |
| Jacklin et al  2003 [13] | Video-consultations between GPs and specialists | RCT (CCA) | SF-12, patient satisfaction and ability to cope | Direct health care costs, travel and time costs for patients | Secondary article, case report notes and official reimbursement fees. | Equal effectiveness, reduced costs for the patients and increased costs for the health system |  | |
| Jansa et al 2006 [14] | Home monitoring in diabetes care | RCT (CMA) | Blood glucose level, hypoglycaemic events, DiaQoL, SF-12, knowledge questionnaire and self-management | Direct health care costs, travel and time costs for patients | Questionnaires, case notes, medical costs not reported. | Similar results in effectiveness and health care costs, reduced patient costs |  | |
| Jerant et al 2001 [15] | Home care via TM for chronic heart failure (CHF) | RCT (CCA) | SF-36 and a specific heart failure questionnaire, patient satisfaction | Direct health care costs | Observed in the study, case notes, questionnaires and official databases | No difference in effectiveness, reduced ED-visits for TM group, and no difference in readmission or other health care visits |  | |
| Johnston B et al. 2000 [16] | Home care via TM for patients with chronic diseases | RCT (CCA) | SF-12 at baseline, patient satisfaction and quality of care (medication compliance, knowledge and self care) | Direct health care costs | Case notes, patient records, patient interviews and databases | No difference in the quality indicators and TM has potential to save costs. |  | |
| Johnston K et al. 2004 [17] | Eye examination via VC between South Africa and UK | Case series; one group (CUA) | Improved visual acuity and DALYs | Direct health care costs | Expert opinions and literature; cost observed in the study and local sources | TM costs £53/DALY and is within cost-effective range |  | |
| Kildemoes and Kristiansen 2004 [18] | Pre-hospital diagnostics in emergency care (AMI) via TM | Decision analytic modelling (CEA) | LYG | Direct health care costs, limited information provided | Published studies, Swedish cost data, some costs based on rough estimates | TM not justified; ICER was DKK854 700 |  | |
| Kortke et al, 2006 [19] | Rehabilitation at home via monitoring after cardiac surgery | Non-randomised trial with two groups (CCA) | SF-36, ECG and pulse, physical capacity (spiroergometry), body mass index | Direct health care costs, travel costs for patients | Questionnaires and case notes, costs not reported | No difference in the effectiveness except for fewer episodes of angina in the TM group and TM costs less |  | |
| Mason et al 2006 [20] | Diabetes home care via TM | RCT and modelling (CUA) | Blood glucose level and QALYs | Direct health care costs, no costs of usual care | Appears to have been observed in the study, published studies and national references prices | TM were found to be borderline cost-effective, ICER amounted to a total of £43 400/QALY |  | |
| Modai et al. 2006 [21] | VC in psychiatry | Trial-based with matched controls  (CCA) | Adherence as mean visits/mean missed visits, hospitalisation days, safety measured on a rating scale, patient satisfaction | Direct health care and patient travel costs, limited details provided | Not reported | Adherence better for TM group at a higher cost |  | |
| Morrison et al 2001 [22] | Home surveillance  of preterm labour | Case-control study  (CCA) | Gestational age at delivery, birth weight, no. of caesareans, LOS for the infants | Direct health care costs, limited information provided | HMO databases and clinical databases | TM improved clinical outcome and reduced costs |  | |
| Noble et al. 2005 [23] | VC for minor injuries at peripheral emergency unit | RCT (CCA) | Diagnostic safety and return to ADL | Direct health care costs, travel and time costs for patients and their family | Secondary article, costs observed in study | Similar effectiveness and TM is more costly both for patients and the NHS |  | |
| Noel et al. 2004 [24] | Home care for elderly with complex conditions via TM | RCT (CCA) | Blood glucose level, measures of cognitive status, functional level, self-rated health status and QoL, patient satisfaction | Direct health care costs and patient travel costs | Questionnaires and health provider’s databases | No difference in effectiveness except for cognitive status which improved more for TM-group, the costs decreased for both groups |  | |
| Pelletier-Fleury et al 2001 [25] | Home monitoring of sleep apnea | Cross-over study (CMA) | Recording legibility criterion (effectiveness in making diagnoses) | Direct health care costs | Observed in the study, hospital billing system and official prices | Similar effectiveness at an increased costs |  | |
| Rendina 1998 [26] | Peadiatric cardiology  examinations via TM | Before and after study with controls (CCA) | Mortality, LOS and patient transfers | Direct hospital costs (no staff costs) | Neonatal Data Management System, medical records logbooks and financial service department | No difference in effectiveness and reduced costs |  | |
| Ruskin et al. 2004 [27] | VC in psychiatry | RCT (CCA) | Scales for depression, anxiety and functioning, SF-12 and treatment adherence | Direct health care costs, incl. specialist travel costs | Observed in the study and financial systems (DRGs) | No differences in effectiveness and TM more expensive |  | |
| Santamaria et al 2004 [28] | Wound care via TM | RCT (CCA) | Percentage change in wound size, number of amputations and deaths | Direct health care costs; no actual costs for the individual subjects | International data, hospital financial systems (DRGs) and author’s assumptions | Improved effectiveness; increased healing rate, fewer amputations and lower costs. |  | |
| Scalvini et al 2005 [29] | Cardiology  examinations via TM for patients with CHF | Two groups no information on controls (CCA) | Minnesota Living Questionnaire, number of patients with instability and deaths | Direct health care costs | Questionnaires, cost data not reported | The quality indicators increased for the TM-group and costs less |  | |
| Smith et al. 2002 [30] | Home monitoring of sleep apnea | Before and after study (CCA) | Agreement in reading of transmitted data | Direct health care costs | Observed in the study, cost data not reported | TM is feasible and reduced the costs |  | |
| Whited et al 2003 [31] | TM consult-system in dermatology | Decision-analytic modelling (CEA) | Median time to initial definitive intervention | Direct health care costs and patient travel costs. Production loss incl. in sensitivity analysis | Observed in the study, not reported for all cost items | TM decreased the time to intervention but increased the costs |  | |
| Whited et al 2005 [32] | Examination via TM to detect diabetic retinopathy | Decision-analytic modelling (CEA) | Number of true positives detected, laser treatments and severe vision loss averted | Direct health care costs | Published studies, expert opinions, administrative data and market prices | TM was less costly and more effective in 7 out of 9 models |  | |
| Willems  et al. 2007 [33] | Home monitoring of asthmatics | RCT (CUA) | QALYs based on EQ-5D and SF-6D | Direct health care costs, travel and time costs for patients and their family | Hospital accounting system, project costs diaries and Dutch manuals for cost research | TM of limited cost-effectiveness; €31 000/QALY gained for adults and €59 000/QALY gained for the children |  | |

TM: telemedicine; VC: videoconferencing; CHF: chronic heart failure; AMI: acute myocardial infarct; CEA: cost-effectiveness analysis;

CMA: cost-minimisation analysis; CUA: cost-utility analysis; CCA: cost-consequence analysis; QALYs: quality adjusted life-years; LYG: life years gained; LOS: length of stay;

DALYs: disability-adjusted life-years; QoL; quality of life; ADL: activity of daily living; ICER: incremental cost-effectiveness ratio.

The table is adapted from Sculpher & Price (2005)

**List of papers reviewed**

1. Aoki N, Dunn K, Fukui T, Beck JR, Schull WJ, Li HK: **Cost-effectiveness analysis of telemedicine to evaluate diabetic retinopathy in a prison population.** *Diabetes Care* 2004, **27:**1095-1101.

2. Auerbach H, Schreyogg J, Busse R: **Cost-effectiveness analysis of telemedical devices for pre-clinical traffic accident emergency rescue in Germany.** *Technol Health Care* 2006, **14:**189-197.

3. Barnett TE, Chumbler NR, Vogel WB, Beyth RJ, Ryan P, Figueroa S: **The cost-utility of a care coordination/home telehealth programme for veterans with diabetes.** *J Telemed Telecare* 2007, **13:**318-321.

4. Biermann E, Dietrich W, Rihl J, Standl E: **Are there time and cost savings by using telemanagement for patients on intensified insulin therapy? A randomised, controlled trial.** *Comput Methods Programs Biomed* 2002, **69:**137-146.

5. Bohnenkamp SK, McDonald P, Lopez AM, Krupinski E, Blackett A: **Traditional versus telenursing outpatient management of patients with cancer with new ostomies.** *Oncol Nurs Forum* 2004, **31:**1005-1010.

6. Breslow MJ, Rosenfeld BA, Doerfler M, Burke G, Yates G, Stone DJ, Tomaszewicz P, Hochman R, Plocher DW: **Effect of a multiple-site intensive care unit telemedicine program on clinical and economic outcomes: an alternative paradigm for intensivist staffing.** *Crit Care Med* 2004, **32:**31-38.

7. Castillo-Riquelme MC, Lord J, Moseley MJ, Fielder AR, Haines L: **Cost-effectiveness of digital photographic screening for retinopathy of prematurity in the United Kingdom.** *Int J Technol Assess Health Care* 2004, **20:**201-213.

8. Dawson A, Cohen D, Candelier C, Jones G, Sanders J, Thompson A, Arnall C, Coles E: **Domiciliary midwifery support in high-risk pregnancy incorporating telephonic fetal heart rate monitoring: a health technology randomized assessment.** *J Telemed Telecare* 1999, **5:**220-230.

9. Dowie R, Mistry H, Young TA, Weatherburn GC, Gardiner HM, Rigby M, Rowlinson GV, Franklin RC: **Telemedicine in pediatric and perinatal cardiology: economic evaluation of a service in English hospitals.** *Int J Technol Assess Health Care* 2007, **23:**116-125.

10. Eron L, Marineau M: **Treating Infections in the Home Yields Clinical, Economic Benefits.** *DRUG BENEFIT TRENDS* 2007, **19:**109.

11. Ferris DG, Bishai DM, Litaker MS, Dickman ED, Miller JA, Macfee MS: **Telemedicine network telecolposcopy compared with computer-based telecolposcopy.** *J Low Genit Tract Dis* 2004, **8:**94-101.

12. Finkelstein SM, Speedie SM, Potthoff S: **Home telehealth improves clinical outcomes at lower cost for home healthcare.** *Telemed J E Health* 2006, **12:**128-136.

13. Jacklin PB, Roberts JA, Wallace P, Haines A, Harrison R, Barber JA, Thompson SG, Lewis L, Currell R, Parker S, Wainwright P: **Virtual outreach: economic evaluation of joint teleconsultations for patients referred by their general practitioner for a specialist opinion.** *BMJ* 2003, **327:**84.

14. Jansa M, Vidal M, Viaplana J, Levy I, Conget I, Gomis R, Esmatjes E: **Telecare in a structured therapeutic education programme addressed to patients with type 1 diabetes and poor metabolic control.** *Diabetes Res Clin Pract* 2006, **74:**26-32.

15. Jerant AF, Azari R, Nesbitt TS: **Reducing the cost of frequent hospital admissions for congestive heart failure: a randomized trial of a home telecare intervention.** *Med Care* 2001, **39:**1234-1245.

16. Johnston B, Wheeler L, Deuser J, Sousa KH: **Outcomes of the Kaiser Permanente Tele-Home Health Research Project.** *Arch Fam Med* 2000, **9:**40-45.

17. Johnston K, Kennedy C, Murdoch I, Taylor P, Cook C: **The cost-effectiveness of technology transfer using telemedicine.** *Health Policy Plan* 2004, **19:**302-309.

18. Kildemoes HW, Kristiansen IS: **Cost-effectiveness of interventions to reduce the thrombolytic delay for acute myocardial infarction.** *Int J Technol Assess Health Care* 2004, **20:**368-374.

19. Kortke H, Stromeyer H, Zittermann A, Buhr N, Zimmermann E, Wienecke E, Korfer R: **New East-Westfalian Postoperative Therapy Concept: a telemedicine guide for the study of ambulatory rehabilitation of patients after cardiac surgery.** *Telemed J E Health* 2006, **12:**475-483.

20. Mason JM, Young RJ, New JP, Gibson JM, Long AF, Gambling T, Friede T: **Economic Analysis of a Telemedicine Intervention to Improve Glycemic Control in Patients with Diabetes Mellitus: Illustration of a Novel Analytic Method.** *Disease Management and Health Outcomes* 2006, **14:**377.

21. Modai I, Jabarin M, Kurs R, Barak P, Hanan I, Kitain L: **Cost effectiveness, safety, and satisfaction with video telepsychiatry versus face-to-face care in ambulatory settings.** *Telemed J E Health* 2006, **12:**515-520.

22. Morrison J, Bergauer NK, Jacques D, Coleman SK, Stanziano GJ: **Telemedicine: cost-effective management of high-risk pregnancy.** *Manag Care* 2001, **10:**42-46, 48-49.

23. Noble SM, Coast J, Benger JR: **A cost-consequences analysis of minor injuries telemedicine.** *J Telemed Telecare* 2005, **11:**15-19.

24. Noel HC, Vogel DC, Erdos JJ, Cornwall D, Levin F: **Home telehealth reduces healthcare costs.** *Telemed J E Health* 2004, **10:**170-183.

25. Pelletier-Fleury N, Gagnadoux F, Philippe C, Rakotonanahary D, Lanoe JL, Fleury B: **A cost-minimization study of telemedicine. The case of telemonitored polysomnography to diagnose obstructive sleep apnea syndrome.** *Int J Technol Assess Health Care* 2001, **17:**604-611.

26. Rendina MC: **The effect of telemedicine on neonatal intensive care unit length of stay in very low birthweight infants.** *Proc AMIA Symp* 1998**:**111-115.

27. Ruskin PE, Silver-Aylaian M, Kling MA, Reed SA, Bradham DD, Hebel JR, Barrett D, Knowles F, 3rd, Hauser P: **Treatment outcomes in depression: comparison of remote treatment through telepsychiatry to in-person treatment.** *Am J Psychiatry* 2004, **161:**1471-1476.

28. Santamaria N, Carville K, Ellis I, Prentice J: **The effectiveness of digital imaging and remote expert wound consultation on healing rates in chronic lower leg ulcers in the Kimberley region of Western Australia.** *Primary Intention* 2004, **12:**62-72.

29. Scalvini S, Capomolla S, Zanelli E, Benigno M, Domenighini D, Paletta L, Glisenti F, Giordano A: **Effect of home-based telecardiology on chronic heart failure: costs and outcomes.** *J Telemed Telecare* 2005, **11 Suppl 1:**16-18.

30. Smith CE, Cha JJ, Kleinbeck SV, Clements FA, Cook D, Koehler J: **Feasibility of in-home telehealth for conducting nursing research.** *Clin Nurs Res* 2002, **11:**220-233.

31. Whited JD, Datta S, Hall RP, Foy ME, Marbrey LE, Grambow SC, Dudley TK, Simel DL, Oddone EZ: **An economic analysis of a store and forward teledermatology consult system.** *Telemed J E Health* 2003, **9:**351-360.

32. Whited JD, Datta SK, Aiello LM, Aiello LP, Cavallerano JD, Conlin PR, Horton MB, Vigersky RA, Poropatich RK, Challa P, et al: **A modeled economic analysis of a digital tele-ophthalmology system as used by three federal health care agencies for detecting proliferative diabetic retinopathy.** *Telemed J E Health* 2005, **11:**641-651.

33. Willems DC, Joore MA, Hendriks JJ, Wouters EF, Severens JL: **Cost-effectiveness of a nurse-led telemonitoring intervention based on peak expiratory flow measurements in asthmatics: results of a randomised controlled trial.** *Cost Eff Resour Alloc* 2007, **5:**10.
